# Blood level of cadmium and lead in occupationally exposed persons in Gwagwalada, Abuja, Nigeria

**DOI:** 10.1515/intox-2015-0022

**Published:** 2015-09

**Authors:** Lukman Adewale Alli

**Affiliations:** Department of Medical Biochemistry, Faculty of Basic Medical Science, College of Health Sciences, University of Abuja, Nigeria

**Keywords:** lead, cadmium, occupational exposure, atomic absorption spectrophotometer, heavy metals in blood

## Abstract

This study was designed to assess the blood levels of cadmium and lead in some occupationally exposed individuals and compare the values with non-exposed individuals, with the aim of increasing the awareness of health risk caused by these heavy metals. A total of 120 subjects (64 occupationally exposed and 56 non-exposed subjects) with the age range of 15–40 years were studied in cross-sectional study conducted between September 2012 and February 2013 in Gwagwalada area of Abuja, Nigeria. Blood cadmium and lead were analyzed using an atomic absorption spectrophotometer (AAS). The respective mean blood levels of cadmium and lead were 11.63±1.73 μg/dl and 45.43±6.93 μg/dl in occupationally-exposed subjects, while in non-exposed subjects 2.03±0.55 μg/dl and 12.08±2.87 μg/dl. The results show that occupational exposure increases the blood level of cadmium and lead, which consequently increases the health risk of the exposed individuals.

## Introduction

Human exposure to heavy metals such as cadmium and lead could pose serious health challenges, especially among occupationally-exposed workers. Cadmium is a toxic metal that can be released into the environment from human activities such as the use of cadmium containing batteries and phosphate fertilizers, incineration of electronic wastes and tobacco smoking (WHO, 2007). Cadmium is mainly absorbed by inhalation but there may be also additional intake from eating contaminated food at work due to poor personal hygiene at the place of work. For acute exposure by ingestion, the principal effects are gastrointestinal disturbances such as nausea, vomiting, abdominal cramps and diarrhea (Elinder, 1985). Acute inhalation may lead to respiratory manifestations such as severe bronchial and pulmonary irritation, sub-acute pneumonitis, lung emphysema, and, in the most severe situations, death may occur from pulmonary edema (Lauwerys *et al*., 1982). Chronic obstructive airway disease has been associated with long-term inhalation of cadmium (Nordberg, 2006). By chronic cadmium exposure, effects occur mainly on the kidneys, lungs, and bones (Ogunfowokan *et al*., 2002). A relationship has been established between cadmium air exposure and proteinuria, characterized by an increase in the excretion of low molecular weight proteins, such as β2-microglobulin in urine (Kobayashi *et al*., 2009).

Lead is a soft bluish grey metal used as a component of explosives, batteries, metal products and for coating of containers and pipes to prevent corrosion (Goyer, 1986). Occupations associated with high level of lead exposure include battery manufacturing and recycling, lead smelting and foundries, radiator manufacturing and repair, scrap metal handling, petrol/gasoline dispensation in filling stations, pottery making, etc. (Orisakwe, 2009). Lead can be absorbed into the human body through inhalation of lead dust or through ingestion from tobacco and contaminated food or drinks (Patrick, 2006). Lead ingestion from contaminated food or drinks could occur in occupationally exposed individuals who do not wash their hands before eating at the workplace, thereby swallowing lead particles from hands, lips and food products (Alessio, 1988). Though lead can be absorbed through the respiratory and gastrointestinal system (Mushak, 1991), it is more readily absorbed through the respiratory system, which is a common route of exposure in occupationally exposed individuals (Staudinger, 1998; ATSDR, 1999). Tetraethyl lead, an organic form of lead in petrol/gasoline, can also be readily absorbed into the body through human skin (Kovarik, 2005). In Nigeria leaded gasoline/petrol is one of the major sources of lead in the environment as petrol sold in filling stations may contain lead of 0.65 to 0.74 g/L (Galadima & Garba, 2012). Combustion of leaded petrol in the vehicle engine results in oxidation of the organic lead to lead oxide.

2Pb (C_2_H_5_)_4_ + 27O_2_ → 2PbO + 16CO_2_ + 20H_2_O

The resulting lead oxide reacts with halogen to form lead halides such as PbCl_2_ or PbBr_2_, which escape in to the air through the vehicle exhaust (Galadima & Garba, 2012) and are inhaled by humans.

The acceptable blood level of lead in adult humans is less than 10 μg/dl, while the acceptable blood level of cadmium is 0.03–0.12 μg/dl (WHO, 2006). Acute exposure to lead may lead to symptoms such as irritability, muscle pains or cramps, fatigue, anemia and peripheral neuropathy, loss of libido and impotence, while chronic exposure is associated with hypertension, gout, renal failure and encephalopathy (Herman *et al*., 2007). Blood lead level (BLL) and blood cadmium level (BCL) are reliable indicators of recent lead and cadmium exposure (Usuda *et al*., 2011). Lead can be detected in blood within hours of exposure and can remain detected for at least 4 weeks since the half-life of lead in blood is 35 days (Ehrlich *et al*., 1998).

The objective of this study was to compare the blood levels of cadmium and lead of occupationally exposed (such as fuel attendants, auto mechanics, generator mechanics and battery chargers) with non-exposed individuals within the Gwagwalada Area Council of Abuja, Nigeria. The study was aimed at increasing the awareness of such workers to the risk of heavy metal poisoning associated with their work and thus to minimize their exposure to the risk involved.

## Methods

### Study area

This study was carried out in Gwagwalada area council, which is one of the six area councils in Federal Capital Territory, Abuja, Nigeria. It has a land mass of 1,043 square kilometers and a population of 157,770 as at 2006 census (Irechukwu & Chima, 2012). This population has increased rapidly due to migration and demolition of illegal structures in the FCT. Farming and trading is the main occupation of the local population.

### Study design and sample

This study employed a cross-sectional approach. The study sample consisted of 64 occupationally exposed and 56 non-exposed individuals (Control). The occupationally exposed subjects, consisting of automobile mechanics, generator mechanics, petrol dispensing attendants in filling stations and battery chargers, volunteered to participate in this study. These occupations are among those likely to expose individuals to the heavy metals studied. Workers on part-time duties and those who spent less than six months on the job were excluded from this study. The 56 non-exposed (and non-smoker) subjects were fulltime students, randomly selected from the University of Abuja, Gwagwalada campus. The age range of the subjects was between 15 to 40 years.

A self-developed, semi-structured questionnaire, consisting of 20 questions for the exposed and 15 for the nonexposed were administered during a personal interview with the subjects. The additional five questions for the exposed were to determine their occupational exposure to lead and cadmium. Participants were assured of the anonymity of their identity and responses. Before administering the questionnaire, respondents were interviewed to assess the validity, clarity and relevance of the test.

### Blood collection and storage

Venous blood samples (5 ml) were collected via venipuncture into labeled potassium ethylene diamine tetra-acetic acid (K-EDTA) tubes certified for determination of metals in blood. The labeled K-EDTA anticoagulant tubes containing the blood samples were immediately placed in ice pack at the site of blood collection and transferred into a refrigerator at 4 °C (Cornelis *et al*., 2005). To avoid external contamination of the blood sample, the skin at the site of the venipuncture was thoroughly cleaned with methylated spirit to remove any external traces of cadmium and lead that may have been present on the subject's skin.

### Analysis of blood samples for cadmium and lead

Cadmium and lead were extracted from the blood samples using the conventional wet acid digestion method described by Inyengar *et al*. (2005). Blood sample (1 ml) was mixed with 10 ml of conc. HNO_3_ in a conical flask and heated on a hot plate until the solution turned colorless. The solution was allowed to cool before it was made up to 25 ml with de-ionized water. The digested samples were subjected to elemental analyses using a calibrated Buck 205 flame atomic absorption spectrophotometer (Perkin-Elmer, HGA-2100) at the International Institute of Tropical Agriculture (IITA), Ibadan, Nigeria. Cadmium concentration was measured at 228.8 nm, while lead was measured at 283.3 nm (Inyengar *et al*., 1998).

### Quality control and validation of the digestion method

Contamination of the procedure was prevented during analysis of blood lead and cadmium by ensuring that all glassware, pipette tips and stoppers were acid washed (10% nitric acid) and rinsed in distilled ionized water before they were used for procedures in this study.

Validation of the digestion method used and certification of the instrument was done by carrying out recovery experiment and precision analysis (ICH, 1994; Ibeto & Okoye, 2009).

For the recovery experiment, 2 ml of blood sample were measured in duplicate into conical flasks (each sample had two portions of 2 ml). One ml of prepared 1 μg of cadmium and lead standard solution was added to spike one set of the blood samples in 3 conical flasks while the other 3 sets were left unspiked. Perchloric acid and nitric acid were added into all the conical flasks in the ratio of 1:3 (2 ml of perchloric acid and 6 ml of nitric acid). The mixture in the conical flasks was covered and digested until a clear solution was obtained. This was made up to 20 ml after digestion and the concentration of cadmium and lead was determined using an atomic absorption spectrophotometer at 228.8 nm and 283.3 nm, respectively. The percentage recovery was calculated using the formula

%Recovery=(X−Y)/Z×100

X = concentration of cadmium/lead determined in spiked samples

Y = concentration of cadmium/lead determined in unspiked samples

Z = concentration of cadmium/lead added to the spiked samples

The acceptable recovery percentage range was 80–110% for the analyte level of 1 μg/ml (Huber, 1998).

### Precision analysis

1 ml of 1 μg/ml of cadmium and lead standard solution was transferred into a 20 ml conical flask and made up to mark with deionized water to produce 0.05 μg/ml cadmium and lead. This was analyzed 5 times for cadmium and lead.

### Calibration curve

Calibration standard solutions (0.5, 1.0, 2.0, 4.0μg/ml) were prepared separately for cadmium and lead by serial dilution of their stock solutions. The absorbance (corrected for blank) of the standard solutions was measured and plotted against their corresponding concentrations (ICH, 1994; Musa *et al*., 2011).

### Informed consent and ethical approval

Informed consent was obtained from all participants in this study and ethical approval was obtained from the Ethical Committee of the University of Abuja Teaching Hospital, Gwagwalada, Abuja. Procedures carried out in this research were in accordance with the declaration of Helsinki (1964).

### Statistical analysis

The data obtained were analyzed using SPSS 10.00 statistical package. Values are expressed as mean±standard deviation (SD). Student's *t* test was carried out and statistical significance was determined at *p*<0.05.

## Results

The demographic characteristics of the subjects are shown in Table 1. The mean age of the subjects was 27.5±9.5 yr. Six (9.4%) of the occupationally exposed subjects were females and the majority (60%) of the workers had secondary level of education. The auotomobile mechanics constituted the highest percentage (46.9%) of the study population. A large percentage (70%) of the workers were cigarette smokers, which could constitute an additional risk of exposure to cadmium. The mean blood cadmium level (BCL) and mean blood lead level (BLL) for the occupationally exposed population was 11.63±1.73 μg/dl and 45.43±6.93 μg/dl, respectively, while in the non-exposed population, BCL and BLL were 2.03±0.55 μg/dl and 12.08±2.87 μg/dl, respectively (Table 2). The age group of 31–40 yr had the highest value of BCL and BLL of 12.56±2.40 μg/dl and 48.95±7.35 μg/dl, respectively (Tables 2 and 3).

**Table 1:** Demographic characteristics of study subjects.

Characteristics	Exposed workers		Control subjects	
	N	%	N	%
Sex			
Male	58	90.6	41	73.2
Female	6	9.4	15	26.8
Total	64	100	56	100
Age			
15–20	9	14.0	14	25
21–30	38	59.4	36	64.3
31–40	17	26.6	6	10.7
Total	64	100	56	100
Educational status			
No formal education	2	3.1	–	–
Primary level education	15	23.4	–	–
Secondary level education	38	59.4	–	–
Tertiary level education	9	14.1	56	100
Total	64	100	56	100
Occupation			
Automobile mechanics	30	46.9	–	–
Generator mechanics	13	20.3	–	–
Fuel attendants	12	18.7	–	–
Others (battery charger, spray painter)	9	14.1	–	–
Total	64	100	–	–
Associated risk factors			
Cigarette smoking			
YES	42	70.0	–	–
NO	18	30.0	–	–
Hand washing before eating at work			
YES	42	70.0	–	–
NO	18	30.0	–	–

**Table 2:** Comparison of mean blood cadmium level within age groups.

Age group (Year)	Mean blood cadmium level (μg/dl)	
	Occupationally exposed	Non-exposed
15–20	10.58±1.25*	1.55±0.35
21–30	11.75±1.55*	1.74±0.55
31–40	12.56±2.40*	2.80±0.75

* Significantly different from control at *p*<0.05

**Table 3:** Comparison of mean blood lead level within age group.

Age group (Year)	Mean blood lead level (μg/dl)	
	Occupationally exposed	Non-exposed
15–20	41.70±6.54*	11.40±2.45
21–30	45.65±6.90*	12.25±2.65
31–40	48.95±7.35*	12.60±3.50

* Significantly different from control at *p*<0.05

The spray painters and battery chargers had the highest mean BCL of 12.85±1.45 μg/dl, while automobile mechanics had the highest level of BLL of 49.55±7.40 μg/dl. The lowest BCL, 10.46±1.05 μg/dl, was observed in fuel attendants, while the lowest BLL, 41.38±6.35 μg/dl, was recorded in generator mechanics (Table 4). The average number of years at work for the battery chargers and spray painters was 14 years and the average daily hours of exposure were 9 hours. The automobile mechanics recorded an average of 10 hours daily and an average of 6 years at work.

**Table 4:** Mean blood cadmium and lead level in the occupationally exposed.

Occupation	Mean blood level (μg/dl)		Average number of years spent on job	Average number of hours spent on job
	Cadmium	Lead		
Automobile mechanics	11.75±1.25	49.55±7.40	6	10
Generator mechanics	11.95±1.15	41.38±6.35	13	9
Fuel attendants	10.46±1.05	48.45±7.25	2	10
Others (Battery charger, spray painter)	12.85±1.45	45.38±6.95	14	9

## Discussion

Sixty-four occupationally exposed individuals with a minimum of one year experience on the current job and fifty-six non-occupationally exposed individuals were recruited for this research.

There was a significant difference in the mean blood Cd concentrations between the occupationally exposed and non-exposed subjects in this study. The mean blood Cd concentration for the occupationally exposed group (11.63±1.73 μg/dl) is significantly higher than in the nonexposed group (2.03±0.55μg/dl). Even the least cadmium level in the occupationally exposed (10.58±1.25 μg/dl) was higher than the maximum cadmium level in the nonexposed (2.80±0.75 μg/dl). The mean blood cadmium level observed in this study was lower than the 48.8±5.1 μg/dl reported by Ibeto and Okoye (2009) for blood cadmium level of occupationally exposed individuals within Enugu, Nigeria. It was however higher than the 2.29±0.12 μg/dl reported by Musa *et al*., (2011) for blood cadmium level in occupationally exposed individuals in Zaria, Nigeria.

The mean blood cadmium level for both the occupationally exposed and non-occupationally exposed subjects in this study were higher than the WHO’s permissible range of 0.03–0.12 μg/dl of Cd (WHO, 1996). The high blood cadmium level may accumulate and bind to metallothionein in the liver and kidney (Usuda *et al*., 2011). Clinical signs of cadmium toxicity such as tubular and glomerular damage in the kidney usually manifest as elevated urinary excretion of β2-microglobulin. Damage to the proximal tubule may reduce the conversion of 25-hydroxylcholecalciferol to 1,25 dihdroxylcholecalciferol resulting in reduced bone mineralization and osteomalacia (Jarup *et al*., 1998).

Besides cadmium intake via the gastro-intestinal tract and inhalation from burning of fossil fuel, an additional source of cadmium among the occupationally exposed subjects is cigarette smoking, which was observed to be common among artisans, as one cigarette may contain 1–2 μg of cadmium. It has been estimated that a person smoking 10–20 cigarettes a day will absorb 0.5–1 μg of cadmium (Jarup *et al*., 1998; Usuda *et al*., 2011).

The mean blood lead concentration for the occupationally exposed group (45.43±6.93μg/dl) was significantly higher than the blood lead concentration in non-exposed individuals (12.08±2.87μg/dl). This result is slightly lower than 48.5±9.08 μg/dl recorded for occupationally exposed workers in Abeokuta, Nigeria, by Babalola *et al*. (2005). It is however higher than the mean blood lead level of 18.1 μg/dl±6.4 and 10.2 μg/dl±2.7 reported by Ogunsola *et al*. (1994) for occupationally exposed traffic wardens in Lagos and Ife, Nigeria, respectively. It is also higher than 12.3 μg/dl reported by Omokhodion (1994) for the male population in Ibadan, Nigeria. Considering the fact that the half-life of lead in the blood is 35 days, the mean blood level observed in this study is suggestive of recent lead exposure. The continuous use of leaded gasoline for vehicles in Nigeria (Omokhodion, 1994; Musa *et al*., 2011) may contribute to the relatively high blood level of lead among automobile mechanics and fuel attendants when compared to other groups in this study. Poor work habits observed among the automechanics, involving sucking of petrol/gasoline with the mouth while working, could also contribute significantly to the level of lead observed in their blood. Other sources of lead (among exposed and non-exposed individuals) in this study include indiscriminate open air burning of garbage, which may contain used batteries, discarded tires, industrial/agricultural wastes and other lead products. The high blood lead levels observed in the non-occupationally exposed in this study and in several other studies (Ogunfowokan *et al*., 2002; Orisakwe, 2009; Galadima & Garba, 2012) are indicative of the extent of environmental lead pollution in Nigeria. This is of serious concern as it implies that many Nigerians are exposed to lead from the environment irrespective of their occupation.

The average lead value for the exposed and nonexposed subjects in this study was higher than the WHO permissible range of less than 10 μg/dl of lead in adults, while no safe blood lead threshold has been identified for children (CDC, 2012).

The mean BCL and BLL of the occupationally-exposed subjects correlated with age, with the younger age group recording lower values for BCL and BLL. However, the average number of years spent on the job failed to have a significant effect on the mean BLL and BCL.

## Conclusion

The observed blood levels of cadmium and lead in occupationally exposed and non-exposed subjects in this study were above the permissible range and could be a potential health hazard. These concentrations are higher than the permissible range of 0.03–0.12 μg/dl for cadmium (Cd) and 0–10 μg/dl for lead (Pb) (WHO, 1996).

We hereby recommend the use of protective face masks by workers while on duty to prevent inhalation of heavy metal fumes. Also occupationally exposed workers should wash their hands and face before eating at the workplace, shower at work before going home and avoid wearing work clothes in their homes.
